# Clinical outcomes and case volume effect of transanal total mesorectal excision for rectal cancer: a systematic review

**DOI:** 10.1007/s10151-016-1545-0

**Published:** 2016-11-16

**Authors:** C. L. Deijen, A. Tsai, T. W. A. Koedam, M. Veltcamp Helbach, C. Sietses, A. M. Lacy, H. J. Bonjer, J. B. Tuynman

**Affiliations:** 1 Department of Surgery, VU University Medical Centre, PO Box 7057, 1007 MB Amsterdam, The Netherlands; 2 Department of Surgery and Cancer, Imperial College London, London, UK; 3 Department of Surgery, Gelderse Vallei Hospital Ede, Ede, The Netherlands; 4 Department of Surgery, Hospital Clinic de Barcelona, Barcelona, Spain

**Keywords:** Rectal cancer, Total mesorectal excision, Morbidity, Transanal, Case volume

## Abstract

Transanal total mesorectal excision (TaTME) has been developed to improve quality of TME for patients with mid and low rectal cancer. However, despite enthusiastic uptake and teaching facilities, concern exists for safe introduction. TaTME is a complex procedure and potentially a learning curve will hamper clinical outcome. With this systematic review, we aim to provide data regarding morbidity and safety of TaTME. A systematic literature search was performed in MEDLINE (PubMed), EMBASE (Ovid) and Cochrane Library. Case reports, cohort series and comparative series on TaTME for rectal cancer were included. To evaluate a potential effect of case volume, low-volume centres (*n* ≤ 30 total volume) were compared with high-volume centres (*n* > 30 total volume). Thirty-three studies were identified (three case reports, 25 case series, five comparative studies), including 794 patients. Conversion was performed in 3.0% of the procedures. The complication rate was 40.3, and 11.5% were major complications. The quality of the mesorectum was “complete” in 87.6%, and the circumferential resection margin (CRM) was involved in 4.7%. In low- versus high-volume centres, the conversion rate was 4.3 versus 2.7%, and major complication rates were 12.2 versus 10.5%, respectively. TME quality was “complete” in 80.5 versus 89.7%, and CRM involvement was 4.8 and 4.5% in low- versus high-volume centres, respectively. TaTME for mid and low rectal cancer is a promising technique; however, it is associated with considerable morbidity. Safe implementation of the TaTME should include proctoring and quality assurance preferably within a trial setting.

## Introduction

Transanal total mesorectal excision (TaTME) has had tremendous attention since its introduction in 2010 by the group of Lacy [1]. The TaTME technique has been developed to improve the quality of the TME procedure for patients with mid and low rectal cancer. In TaTME, the low pelvic mesorectum is approached through the anus using a laparoscopic single-port platform. Potentially, TaTME facilitates the quality of dissection and decreases the need for definitive colostomies and conversions to open technique. Moreover, the TaTME technique aims to achieve higher rates of complete specimens, better visual determination of the distal margin and lower rates of involved circumferential resection margin (CRM) compared to abdominal TME. Especially in low rectal cancer surgery, relative higher rates of incomplete specimens and higher rates of CRM involvement have been reported compared to tumours located in the upper rectum [2–11]. Mid and low rectal cancer are associated with worse outcome when compared to high rectal cancer due to the difficult access of the lower pelvis. The innovative TaTME technique has the potential to improve these results. However, randomised clinical trials evaluating this new technique are lacking [12–14].

Despite the potential benefits, concern exists for uncontrolled widespread adaptation. TaTME is a complex procedure and a learning curve might influence initial clinical results. Since poor surgical quality in rectal cancer is associated with poor outcome, quality assurance of the new surgical technique seems plausible. Early adaptors of the technique have shown favourable results, but new serious complications have also been published [15–18]. Urethra injury or pelvic side wall injury with bleeding and nerve damage has not been documented for the conventional low anterior resection (LAR) [2–11]. In addition, increased bacterial load as is observed after TaTME might induce the occurrence of presacral abscesses [19]. Most importantly, data regarding oncological outcome after TaTME for mid and low rectal cancer are still scarce [12–18]. Although the aim is to perform resection with intact specimen, rectal wall perforations are observed which can potentially result in tumour spill [1, 15]. Concern exists if luminal contamination with tumour cells of the pelvis results in more recurrences despite a negative resection margin and good quality specimen. In addition to oncological outcome, the long-term functional outcome of the procedure has to be awaited. Potentially, lower anastomosis results in worse functional outcome compared to abdominal laparoscopic TME.

With this systematic review, we aim to provide a comprehensive overview of the current data regarding safety of the TaTME procedure reporting on perioperative and oncological results with specific focus on adverse events and outcomes.

## Materials and methods

### Search strategy

This systematic review was conducted according to the Preferred Reporting Items for Systematic Reviews and Meta-Analysis (PRISMA) guidelines [20]. MEDLINE (PubMed), EMBASE (Ovid) and the Cochrane Library were searched systematically. The search period was from January 1 2005 until July 1 2016. The following search terms were used: (((excision*[tiab] OR resection*[tiab] OR TME[tiab] OR TaTME[tiab] OR TAMIS[tiab] OR NOTES[tiab] OR proctectom*[tiab]) AND (transanal*[tiab] OR trans-anal*[tiab])) OR ((excision*[ot] OR resection*[ot] OR TME[ot] OR TaTME[ot] OR TAMIS[ot] OR NOTES[ot] OR proctectom*[ot]) AND (transanal*[ot] OR trans-anal*[ot]))) AND ((((“Neoplasms”[Mesh] OR neoplas*[tw] OR tumor*[tw] OR tumour*[tw] OR cancer*[tw] OR malignan*[tw] OR oncolog*[tw] OR carcinom*[tw] OR adenocarcinom*[tw]) AND (“Rectum”[Mesh] OR rectum[tiab] OR rectal[tiab] OR colorect*[tiab] OR mesorect*[tiab])) AND (“surgery”[Subheading] OR surgery[tiab] OR surgical[tiab] OR operati*[tiab])) OR (“Rectal Neoplasms/surgery”[Mesh:noexp])). References of the retrieved papers were screened to search for additional reports.

### Inclusion and exclusion criteria

Published clinical studies on TaTME for rectal cancer reporting clinical and pathological outcomes were included. Case reports, cohort series and comparative series were eligible. Abstracts, reports with no peer-reviewed data and reports on robotic TaTME were excluded. No restriction was made based on included number of patients. Only articles in European languages were included. Two reviewers independently assessed all titles, abstracts and full texts for potential inclusion. When required, a third reviewer was consulted. Included articles based on full text were checked for overlapping data with other studies. Studies with potential overlapping patient populations were excluded for the overall analysis and subanalysis regarding volume.

### Endpoints and data extraction

The primary endpoints of this study were short-term morbidity and specimen outcome. The following data were collected from included studies: first author, year of publication, number of patients, patient and tumour characteristics (gender, BMI, age, ASA classification, tumour distance, clinical TNM stage, neoadjuvant therapy), surgical details (operative time, type of anastomosis, use of diverting ileostomy, approach with synchronous abdominal and transanal resection, intraoperative complications, conversion rate), pathology outcomes (TME quality, involvement of CRM, involvement of distal resection margin, pathological T and N stage) and post-operative outcomes (hospital stay, post-operative complications, 30-day mortality rate and local and distant recurrence rates after follow-up of 12 months).

Heterogeneity in data on the height of tumour restricted data evaluation. Therefore, height was adjusted using international accepted definitions for anal verge (baseline 0 cm), dentate line (+1.9 cm) and anorectal junction (+4 cm) [21–23]. Post-operative complications were reported as classified by Clavien–Dindo [24]. Minor complications were defined as complications needing non-invasive treatment (Clavien–Dindo classification I or II), and major complications were defined as complications needing invasive treatment (Clavien–Dindo ≥ III).

### Subanalysis low-volume centres versus high-volume centres

To identify a possible difference in outcome depending on the volume in the TaTME technique, subanalysis of all variables was performed comparing low-volume centres (*n* ≤ 30 total volume) with high-volume centres (*n* > 30 total volume) and excluding potential (partial) duplicates of cases in publications by centres that published multiple cohort series [25].

### Statistical analysis

For all participating patients from the different included studies, data for several variables were pooled in a way as if the patients participated in one study. The mean of the variable of interest of each included study was multiplied with the number of participants in that study, and subsequently, all thus obtained products were added up and divided by the total number of participants in all included studies to obtain a pooled mean. For percentages of dichotomous variables of the different studies, a comparable method was applied. Because of variation in the studies regarding reporting an overall mean or median for the specified endpoint, the mean percentages and weighted means are based on either mean or median of the reporting studies. Furthermore, ranges are used to show the minimum and maximum of the reported means or medians in the different studies. For comparing numeric variables of low- and high-volume centres, an independent *T* test was used. Review Manager version 5.3.5 (2014) was used to calculate the risk difference of dichotomous outcomes of the comparative studies and to make forest plots. To account for clinical heterogeneity, the random effects model based on DerSimonian and Laird’s method were used. A *p* value <0.05 was considered statistically significant.

### Quality assessment: MINORS instrument

Quality assessment of the included articles was performed using the MINORS instrument, an index for the assessment of non-randomised studies [26]. A total of eight items are scored for non-comparative studies and 12 for comparative studies. The items are scored 0 (not reported), 1 (reported but inadequate) or 2 (reported and adequate). The global ideal score is 16 for non-comparative studies and 24 for comparative studies.

## Results

### Included studies

**Fig. 1:**
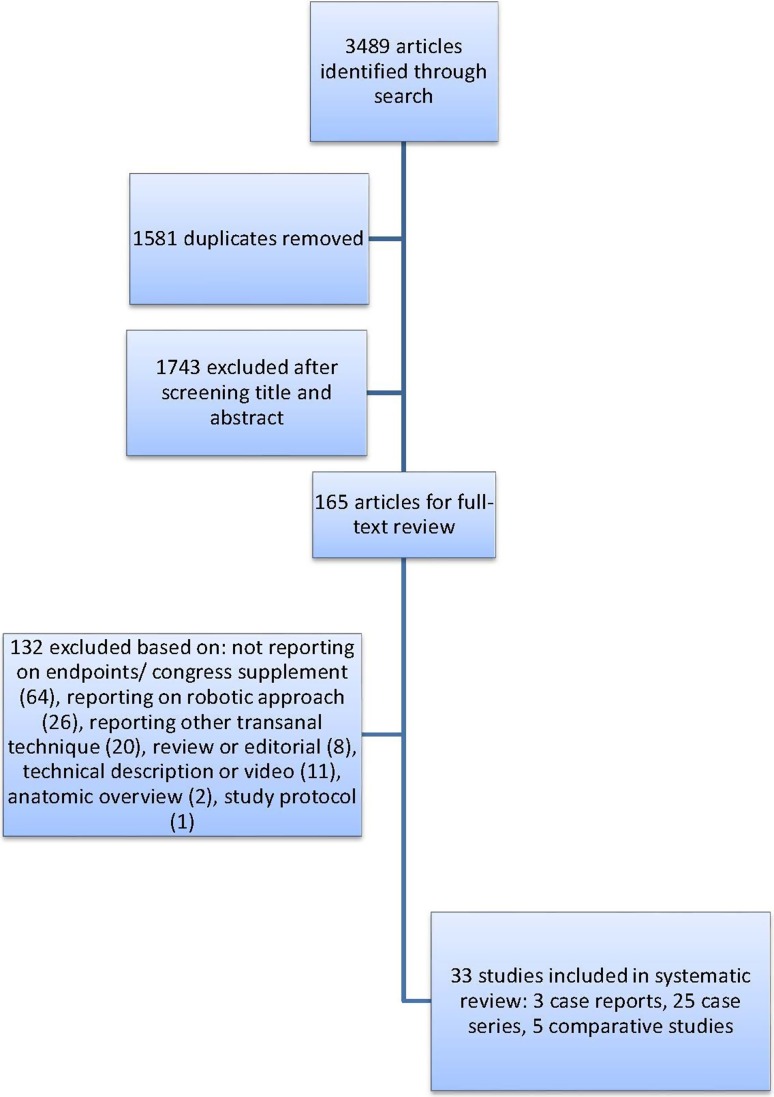
Flow chart of selection process

The literature search identified a total of 3489 articles (EMBASE *n* = 2132, PubMed *n* = 1314 and Cochrane Library *n* = 43). A total of 1581 duplicates were removed, and 1743 articles were excluded after screening title and reading abstract (performed by both CD and AT), leaving 165 articles for full-text review. Finally, 33 articles fulfilled all the inclusion criteria and met no exclusion criteria and were included for analyses [1, 15–18, 27–54]. These 33 articles comprised 3 case reports, 25 case series and 5 comparative studies (Fig. 1). The mean MINORS index of the non-comparative studies was 13 (range 8–15) and of the comparative studies 20 (range 20–21), indicating fair overall quality of the included articles. To correct for overlapping patient populations, 9 of these studies were not included in the overall analysis (Table 1).

**Table 1:** Details of included studies

Author	Year of publication	*N*	Gender M	Gender F	BMI (kg/m^2^)	Age (year)	ASA score (mean)	Tumour distance (cm)^b^
Sylla^a^	2010	1	0	1	20	76	NR	8
Dumont	2012	4	4	0	23.4	66.8	NR	5.3
Zorron^a^	2012	2	1	1	NR	65	1	7
Lacy^a^	2013	3	1	2	21.7	73	NR	9.7
Lacy^a^	2013	20	11	9	25.3	65	2	6.5
Sylla	2013	5	3	2	25.7	48.6	NR	5.7
Velthuis^a^	2013	5	2	3	NR	69.4	NR	6
Rouanet	2013	30	30	0	26	65	NR	5
Zhang	2013	1	0	1	20	48	NR	7
Fernandez-Hevia^a^	2014	37	24	13	23.7	64.5	2	5.8
Velthuis^a^	2014	25	18	7	25	64	NR	8
Atallah^a^	2014	20	14	6	24	57	2	5
Chouillard	2014	16	6	10	27.9	57.7	2	8.4
Meng	2014	3	2	1	NR	80	NR	6.2
Zorron	2014	9	5	4	NR	62.6	1	7.56
Veltcamp Helbach	2015	80	48	32	27.5	66.5	NR	7.2
Tuech	2015	56	41	15	27	65	2	4
Muratore	2015	26	16	10	26.2	65.8	NR	4.4
Elmore	2015	6	2	4	25	61.3	2	5.5
Knol	2015	10	8	2	26.5	60.5	NR	6.89
Serra-Aracil	2015	32	24	8	25	68	2	8
Lacy	2015	140	89	51	25.2	65.5	2	7.6
Perdawood	2015	25	19	6	28	70	2	8
McLemore	2015	1	1	0	32	66	NR	2
Buchs^a^	2015	20	14	6	27.1	59.3	2	6
Chen	2015	50	38	12	24.2	57.3	2	5.8
Prochazka	2015	17	11	6	28	68	3	6.0
Rink	2015	24	18	6	25	57	2	5
Burke	2016	50	30	20	26	56.5	2	4.4
Rasulov	2016	22	11	11	26	56	NR	6.5
Marks	2016	4	1	3	26	56	NR	5.1
Foo	2016	10	5	5	23.4	62.2	2	5.1
Buchs	2016	40	32	8	27.4	64.4	2	7

Author	Year publication	*N*	Conversion (%)	TME quality (%) complete^e^	Positive distal resection margin (%)	CRM involvement (%)	pT3+ (%)	Harvested lymph nodes (N)
Sylla^a^	2010	1	0	100	0	0	0	23
Dumont	2012	4	0	^c^	0	0	NR	16
Zorron^a^	2012	2	0	^c^	0	0	100	11.5
Lacy^a^	2013	3	0	^c^	0	0	66.7	NR
Lacy^a^	2013	20	0	^c^	0	0	NR	15.9
Sylla	2013	5	0	100	0	0	0	33
Velthuis^a^	2013	5	NR	100	0	0	40	12
Rouanet	2013	30	6.7	100	0	0	100	12
Zhang	2013	1	0	100	0	13.3	70	13
Fernandez-Hevia^a^	2014	37	0	91.9	NR	0	62.2	14.3
Velthuis^a^	2014	25	NR	96	NR	4	NR	14
Atallah^a^	2014	20	NR	55	5	5	55	22.5
Chouillard	2014	16	6.3	NR	0	0	50.1	21
Meng	2014	3	0	NR	0	0	66.7	NR
Zorron	2014	9	22	^b^	0	11	66.7	13
Veltcamp Helbach	2015	80	5	88.8	0	2.5	52.5	14
Tuech	2015	56	5.4	83.9	0	5.4	39.3	12
Muratore	2015	26	0	88.5	0	0	30.8	8
Elmore	2015	6	0	100	0	0	50	32
Knol	2015	10	0	90	0	0	40	10.5
Serra-Aracil	2015	32	0	93.8	0	0	NR	15
Lacy	2015	140	0	97.1	0	6.4	NR	14.7
Perdawood	2015	25	0	80	NR	4	68	21
McLemore	2015	1	0	100	NR	NR	0	13
Buchs^a^	2015	20	15	80	0	5.9	25	23.3
Chen	2015	50	2	NR	NR	4	NR	16.7
Prochazka	2015	17	0	47.1	0	11.8	35.3	10
Rink	2015	24	NR	91.67	0	8.3	33.3	14
Burke	2016	50	2.2	72	2	4.0	50	18
Rasulov	2016	22	4	68	NR	5.0	23	17
Marks	2016	4	0	100	0	0.0	25	6
Foo	2016	10	10	60	0	0.0	NR	15.6
Buchs	2016	40	7.5	92.5	0	5.0	32.5	20

Author	Year publication	*N*	Hospital stay (days)	Post-operative complications (%)		30-Day mortality (%)
				Minor^f^	Major^f^	
Sylla^a^	2010	1	4	0	0	0
Dumont	2012	4	13	0	25	0
Zorron^a^	2012	2	6	50	0	0
Lacy^a^	2013	3	4.7	33.3	0	0
Lacy^a^	2013	20	6.5	20	0	0
Sylla	2013	5	5.2	60	0	0
Velthuis^a^	2013	5	NR	40	20	NR
Rouanet	2013	30	14	33.3	13.3	0
Zhang	2013	1	NR	0	0	0
Fernandez-Hevia^a^	2014	37	6.8	24.3	8.1	0
Velthuis^a^	2014	25	NR	NR	NR	NR
Atallah^a^	2014	20	4.5	75	25	0
Chouillard	2014	16	NR	0	18.8	0
Meng	2014	3	6.5	0	0	NR
Zorron	2014	9	7.6	11.1	11.1	0
Veltcamp Helbach	2015	80	8	26.3	12.5	1
Tuech	2015	56	10	19.6	5.4	0
Muratore	2015	26	7	15.4	11.5	3.8
Elmore	2015	6	10.3	0	33.3	0
Knol	2015	10	6	20	0	0
Serra-Aracil	2015	32	8	18.8	25	0
Lacy	2015	140	6	36.4	10	0
Perdawood	2015	25	5	28	24	0
McLemore	2015	1	7	100	100	0
Buchs^a^	2015	20	7	25	10	0
Chen	2015	50	7.4	20	6	0
Prochazka	2015	17	9	23.5	11.8	0
Rink	2015	24	NR	12.5	12.5	0
Burke	2016	50	4.5	28	18	0
Rasulov	2016	22	8	27	0	0
Marks	2016	4	5	25	0	0
Foo	2016	10	6	20	0	0
Buchs	2016	40	7.5	27.5	12.5	0

*BMI* body mass index, *ASA* American Society of Anesthesiologists, *NR* not reported, *TME* total mesorectal excision, *CRM* circumferential resection margin

^a^Potentially overlapping patient population

^b^Measured from anal verge

^c^% of total patients with anastomosis

^d^% of total patients

^e^Defined by Quirke

^f^Minor was defined as Clavien–Dindo classification I or II, and major was defined as ≥III

### Patient and tumour characteristics

In total, 794 patients were included, ranging from 1 patient to 140 patients per study. The tumour distance was measured from the anal verge in 24 studies, in 6 from the anorectal junction and in 3 from the dentate line. With correction for overlapping studies, in total 661 patients were included [444 males (67%) and 217 females (33%)] The calculated distance from the anal verge ranged from 2.0 cm to 8.4 cm with a weighted mean of 6.3 cm. Other baseline and tumour characteristics are shown in Table 2.

**Table 2:** Baseline and tumour characteristics

	Weighted mean	Range
Gender (%)		
Male	67	
Female	33	
BMI (kg/m^2^)	26.1	20–32
Age (years)	63.4	48–80
ASA score (mean)	2	1–3
Tumour distance (cm)^a^	6.3	2–8.4
cT3–T4 (%)	71.6	40–100
Neoadjuvant therapy (%)	72.5	28–100

*BMI* body mass index, *ASA* American Society of Anesthesiologists

^a^Measured from anal verge

### Surgical details

The operative time ranged from 166 to 369 min with a weighted mean of 243.9 min. In nine of the 33 studies, two surgical teams performed the surgery in some or all of the cases: one for the laparoscopic abdominal approach and one for the transanal approach, working simultaneously. For studies reporting on TaTME with two teams, the weighted mean for the operative time was 209.8 min (range 166–369) compared to 264.5 min (range 204–360) with one operating team. Other surgical details are depicted in Table 3.

**Table 3:** Surgical details and clinical outcomes

	Weighted mean	Range
Conversion (%)	3.0	0–22
Post-operative complications (%)		
Minor^a^	28.8	0–100
Major^a^	11.5	0–100
Operative time (min)	243.9	166–369
Coloanal handsewn anastomosis (%)^b^	53.9	0–100
Diverting ileostomy (%)^c^	90.3	25–100
Colostomy (%)^c^	4.7	0–28
Two-team approach (%)	37.5	0–100
Hospital stay (days)	8.4	4.5–14
30-Day mortality (%)	0.3	0–3.8

^a^Minor was defined as Clavien–Dindo classification I or II, and major was defined as ≥III

^b^% of total patients with anastomosis

^c^% of total patients

### Procedure related complications

In 18 studies, no intra-operative complications were reported, in one study no major complications were not reported, and in two studies the number of intra-operative complications was not mentioned. Of the 12 studies that did report intra-operative complications, five patients experienced side wall damage and five patients urethral damage during surgery. In two patients, the urethral lesion was repaired with sutures during the procedure not resulting in any documented problems postoperatively. In one patient, the lesion was managed non-operatively and no long-term sequelae were documented. In the other patients with urethral injury, the repair and outcome were not described. In four of the patients with side wall damage, the lesions were small without major post-operative morbidity and in the other patient outcome was not reported. One study reported early intraperitoneal CO_2_ leakage hampering the procedure. In one case, extensive pneumatosis of the retroperitoneum and mesentery of the small bowel was observed which stopped the procedure but did not result in any post-operative morbidity. One patient experienced an air embolism with temporary oxygen desaturation. In ten patients, bleeding occurred: in five, the source was the pelvic side wall, in three the bleeding was located presacrally, in one patient the bleeding was the result of injury to the iliac vessels, and in another patient the bleeding was located at the left side of the mesorectum. Finally, in one patient intraoperative bladder injury occurred. The defect was closed laparoscopically and treated with a urinary catheter for one week.

### Pathology outcomes

At histopathological examination, different descriptions were used to define the quality of the mesorectum hampering accurate comparison. In the studies using the definition based on Quirke’s classification (*n* = 19), the weighted mean of the quality of the mesorectum was “complete” in 87.6% and “nearly complete” in 10.9%. Positive distal resection margins were found in 0.2% of the patients. The rate of involvement of CRM was 4.7%. In 45.2% of the patients, a pT3 or pT4 tumour was found at pathological examination (Table 4).

**Table 4:** Pathology outcomes and follow-up

	Weighted mean	Range
TME quality (%)^a^		
Complete	87.6	47.1–100
Nearly complete	10.9	0–52.9
Incomplete	1.5	0–18
Distal resection margin involvement (%)	0.2	0–2
CRM involvement (%)	4.7	0–13.3
pT3–T4 (%)	45.2	0–100
Recurrence^b^		
Local (%)	4	0–16.7
Distant (%)	8.1	5.4–14
Follow-up (months)	18.9	15.1–29

TME total mesorectal excision, *CRM* circumferential resection margin

^a^Defined by Quirke

^b^Only > 12 months

### Post-operative outcomes and complications

The duration of hospital stay ranged from 4.5 to 14 days with a weighted mean of 8.4 days. Total complication rate was 40.3%. Complications reported were: anastomotic leak (37), urinary retention and urinary dysfunction (26), ileus (32), obstruction and intestinal occlusion (15), presacral abscess and pelvic sepsis (18), increased ileostomy output (16), blood transfusion (11), anastomotic stricture (11), haemorrhage (6), bleeding (6), (organ cavity) surgical site infection (8), fever (6), intra-abdominal collection (5), colitis after ileostomy closure (4), nosocomial infection (3), pneumonia (3), small bowel laceration (2), rectovaginal fistula (2), resection of ischaemic conduit (2), perianastomotic fluid collections (2), wound infection (2), acute renal failure (1), anastomotic fistula (1), ureteral stent placement (1), ischaemia of the proximal limb of the colon (1), anastomotic sinus (1), superficial necrosis of colostomy (1), early adhesions (1), internal herniation (1), large haematoma (1), cerebral infarction (1), peritonitis (1), pelvic haematoma (2), septic shock (1), inguinal lymphorrea (1), myocardial infarction (1), pulmonary embolism (1), pelvic collection (1), bilateral calf compartment syndrome (1), prolapsing ischaemic anal mucosa (1), perineal wound dehiscence after proctocolectomy (1), gastroparesis (1), necrosis of descending colon due to injury to marginal artery (1), transient paresthesia of both feet due to intraoperative positioning (1), ascites (1), acute post-operative pancreatitis (1), pseudomembranous colitis (1), necrosis of stoma (1) and enterostomy-related other (1). Post-operative complications defined as minor occurred in 29.5% (range 0–100%) and major complications occurred in 11.3% (range 0–100%). Thirty-day post-operative mortality occurred in two patients in two different studies, accounting for a weighted mean of 0.3%. One patient suffered from anastomotic leak and died after re-operation due to septic complications. The other patient died three days after the operation as a result of myocardial infarction (Table 3).

### Long-term oncological outcomes

None of the studies had 3-year complete follow-up to calculate 3-year disease-free survival. Five studies (including 302 patients) reported follow-up of more than 12 months. Overall time of follow-up was 18.9 months. The local and distant recurrence rates were 4.0 and 8.1%, respectively (Table 4). In one of these studies, five local recurrences occurred during the follow-up period of 21 months. Another study reported two local recurrences, as well as three lung metastases at median follow-up of 29 months. Further, ovarian metastases (1) and para-aortic lymph node metastases (1) were reported in another study after a mean follow-up of 23 months. Another study reported one patient with local recurrence, eight patients with systemic recurrences and two patients with local and systemic recurrence at a median follow-up of 15.1 months. Finally, one study reported two patients with local recurrences and seven patients who developed distant metastases at a median follow-up of 15.1 months.

### Comparative studies

**Fig. 2:**
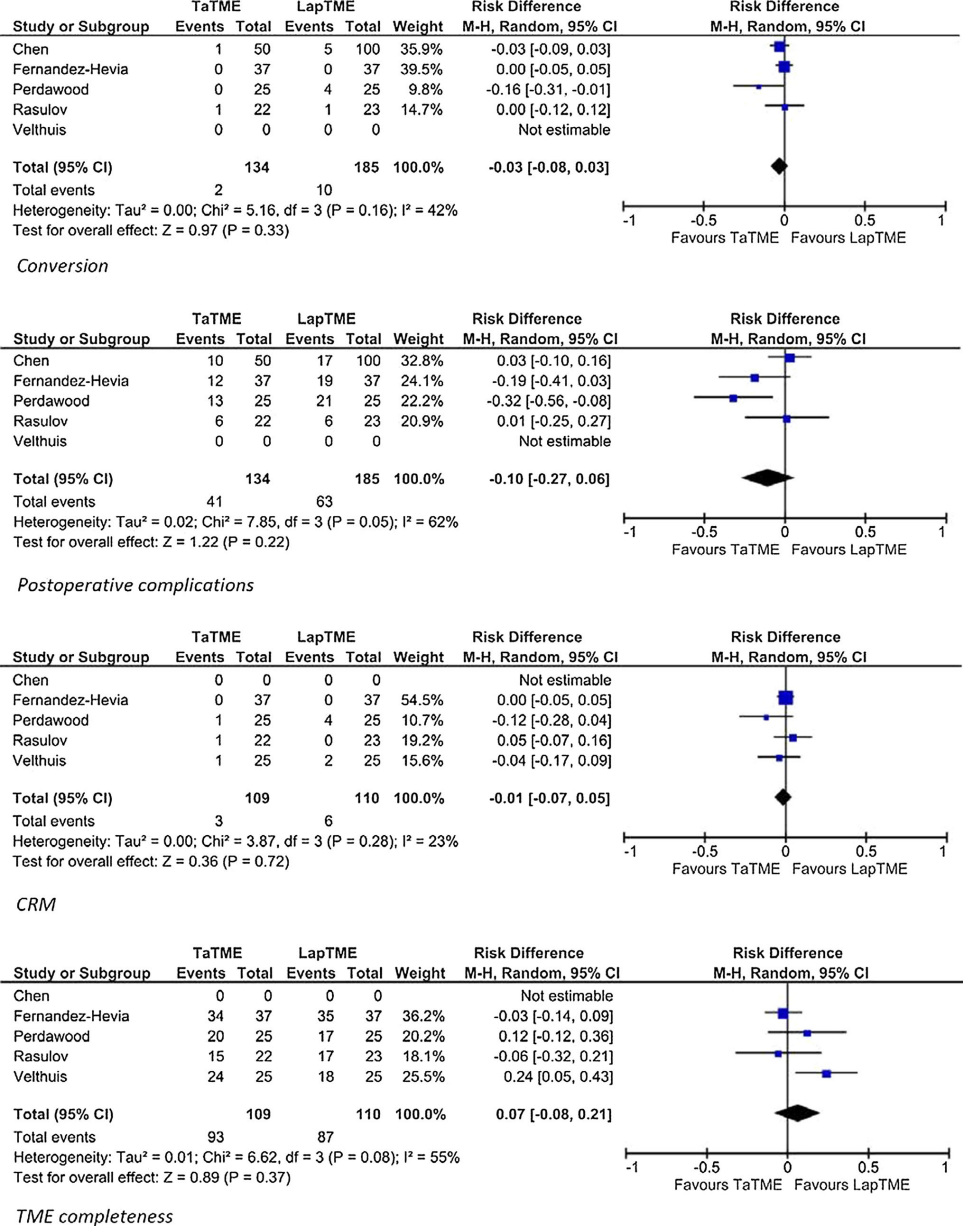
Comparative studies

Five of the included studies evaluated laparoscopic TME versus TaTME in a case-matched study design. Subanalysis of these five studies showed that the weighted means of conversion were 5.4 versus 1.4% for laparoscopic TME and TaTME, respectively. The risk difference of conversion was −0.03 (95% CI −0.08 to 0.03; *p* = 0.33). For post-operative complications, the weighted means were 34.0 versus 30.4%, respectively, with a risk difference of −0.10 (95% CI −0.27 to 0.06; *p* = 0.22). TME completeness was reported in 75.2% in the laparoscopic TME group and 82.8% in the TaTME group. The risk difference was −0.01 (95% CI −0.07 to 0.05; *p* = 0.72). The weighted means of involvement of CRM were 7.6% in the laparoscopic TME group and 3.2% in the TaTME group with a risk difference of 0.07 (95% CI −0.08 to 0.21; *p* = 0.37) (Fig. 2).

### Outcome in low- versus high-volume centres

**Fig. 3:**
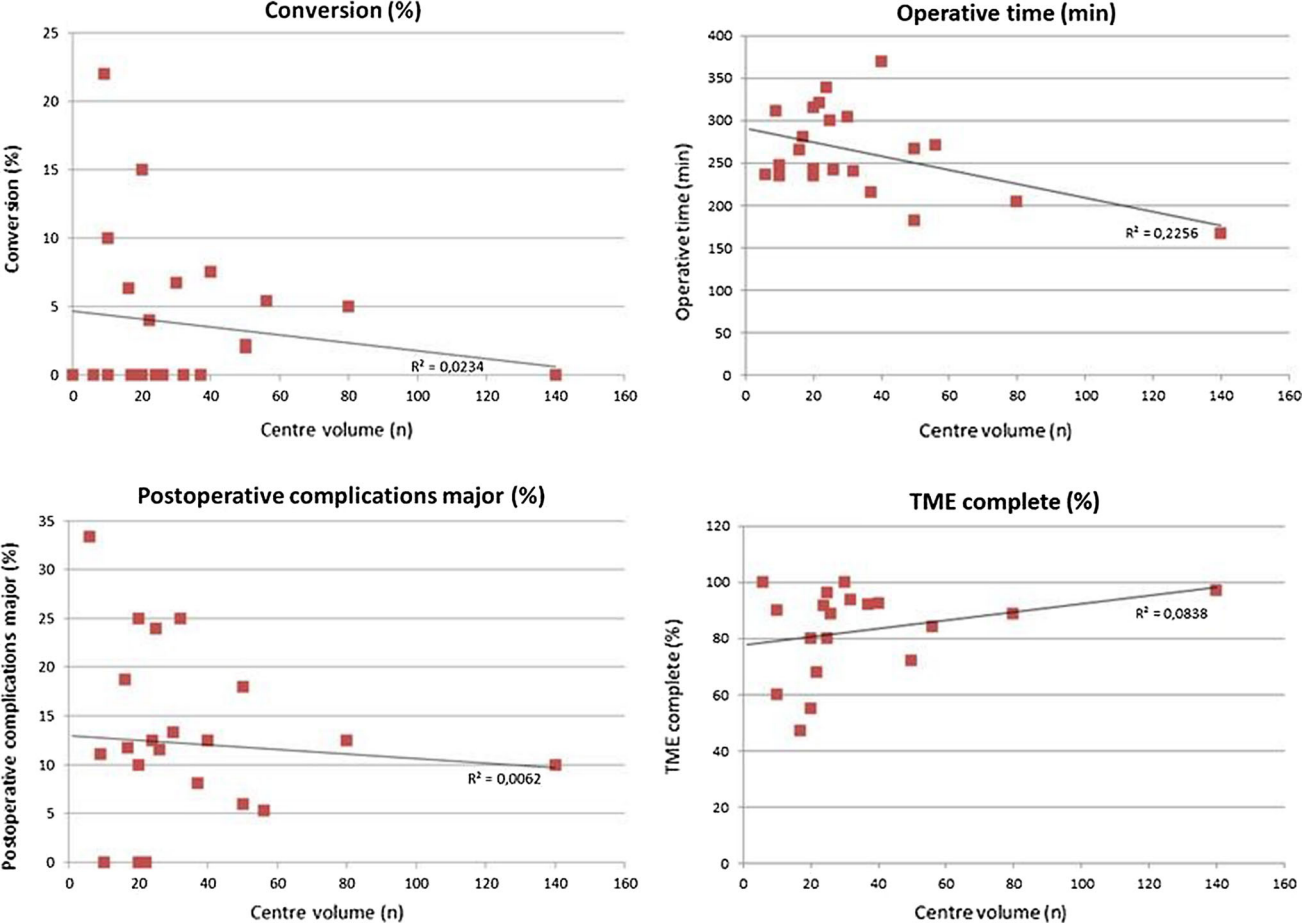
Comparison of low- versus high-volume centres

The centres with a volume of ≤30 patients were compared to the centres with a volume of >30 patients. Regarding surgical details, operative time was shorter in the high-volume centres (222.2 vs. 282.5 min) and the procedure was more often performed with a two-team approach compared to low-volume centres (51.3 vs. 13.7%). Furthermore, the conversion rate was 4.3% in low-volume centres and 2.7% in high-volume centres. The TME quality was more often assessed as “complete” in high-volume centres (80.5 vs. 89.7%), and CRM involvement was 4.8 and 4.5%, respectively. Overall complications were comparable, but the major complication rate was lower in high-volume centres (12.2 vs. 10.5%) (Fig. 3). Long-term oncological data revealed a local recurrence rate of 8.9 versus 2.8% and distant recurrence rate of 7.7 versus 8.1% for the low- and high-volume centres, respectively, although the number of patients with long-term follow-up was limited (Table 5).

**Table 5:** Comparison low- and high-volume centres

	Low-volume centres (*n* ≤ 30) Weighted mean	High-volume centres (*n* > 30) Weighted mean
Conversion (%)	4.3	2.7
Post-operative complications (%): minor^f^	21.9	25.2
Post-operative complications (%): major^f^	12.2	10.5
TME quality (%): complete^d^	80.5	89.7
TME quality (%): nearly complete^d^	15.1	9.0
TME quality (%): incomplete^d^	4.0	1.3
Distal resection margin involvement (%)	0.4	0.3
CRM involvement (%)	4.8	4.5
pT3–T4 (%)	44.3	45.1
Gender M (%)	65.8	67.4
Gender F (%)	34.2	32.6
BMI (kg/m^2^)	26.1	26.0
Age (years)	62.3	63.8
ASA score (mean)	2	2
Tumour distance (cm)^a^	6.0	6.5
cT3–T4 (%)	71.3	69.3
Neoadjuvant therapy (%)	69.8	73.0
Operative time (min)	282.5	222.2
Coloanal handsewn anastomosis (%)^b^	62.6	46.8
Diverting ileostomy (%)^c^	89.8	88.8
Colostomy (%)^c^	6.8	4.8
Two-team approach (%)	13.7	51.3
Hospital stay (days)	6.6	6.5
30-Day mortality (%)	0.4	0.2
Recurrence: local (%)^e^	8.9	2.8
Recurrence: distant (%)^e^	7.7	8.1
Follow-up (months)^e^	21.9	18.3

TME total mesorectal excision, *CRM* circumferential resection margin, *BMI* body mass index, *ASA* American Society of Anesthesiologists

^a^Measured from anal verge

^b^% of total patients with anastomosis

^c^% of total patients

^d^Defined by Quirke

^e^Only > 12 months

^f^Minor was defined as Clavien–Dindo classification I or II, and major was defined as ≥III

## Discussion

This systematic review shows that the TaTME procedure is feasible and safe. The technique is associated with substantial morbidity with comparable rates as reported for laparoscopic abdominal TME. The outcome in terms of specimen quality and CRM rate seems adequate with 87.6 and 4.7%, respectively. In addition, concern exists for the long-term local recurrence rate which is relatively high (4%) despite a relative short follow-up period (18.9 months). Although numbers are insufficient to draw real conclusions yet and no significance was reached, subanalysis from case-matched control studies shows that TME has substantial lower conversion rate compared with the laparoscopic TME group. The weighted mean of the conversion rate in laparoscopic TME was 5.4 versus 1.4% in the TaTME group. Furthermore, specimen completeness was higher in the TaTME group (82.8%) than in the laparoscopic TME group (75.2%) and less patients had involvement of CRM in the TaTME group compared with the laparoscopic group (3.2 vs. 7.6%).

The outcome parameters seem dependent on the volume since small volume centres report longer operation time and higher conversion rate. Furthermore, worse post-operative outcomes (higher colostomy rate, major morbidity, local recurrence rate and lower rate of complete specimens) are observed as compared to the high-volume cohorts.

The total morbidity of the TaTME procedure in this systematic review is comparable with the conventional laparoscopic TME as published in the large randomised trials which display 37–54% total complications [2–11]. Fernandez-Hévia et al. [35] showed a decrease in morbidity including decreased rate of anastomotic leakage compared to conventional TME surgery. This systematic review does not clearly show advantage of the TaTME over the published morbidity rate of LAR. One of the most frequent complications reported was anastomotic leakage which occurred in 37 out of 646 patients with anastomosis (5.7%). The leakage rate compares favourably to reported leakage rates from laparoscopic TME at approximately 10% and this might be an advantage of the TaTME, although randomised data have to be awaited [5]. New possible hazardous complications for TaTME are reported, as urethral lesions and damage of the pelvic side wall which are a concern and need further attention in education. Furthermore, urinary disorders were reported in 26 patients (3.3%) and pelvic abscesses/sepsis in 18 out of 794 patients (2.3%). The reported incidence of presacral abscesses was not increased compared to the abdominal TME procedure. This is unexpected since it has been shown that increased bacterial load is present in the pelvis after TaTME [19]. The low rate of conversions compared to reported laparoscopic TME seems a major improvement and might be accounted as a benefit of TaTME. The reported colostomy rate is very low, but no conclusions can be drawn since considerable selection bias is present since cohort studies do not present intention-to-treat results.

Another potential advantage of the TaTME is improvement in oncological outcome. Surgical specimen quality defined by (1) mesorectal completeness, (2) CRM and (3) distal margins has been shown to be the most important prognostic factor predicting local recurrences [55]. Due to better visualisation in the deep pelvis, meticulous resection can be performed. Cohort and case series of TaTME for rectal cancer included in this systematic review have shown that 2.2% of the specimens were judged as incomplete. In 87% of the cases, the resected specimens were considered intact. In two of the largest randomised trials concerning laparoscopic rectal cancer surgery, the reported rates of complete specimens were 72 and 88% [2, 5]. Another potential improvement in oncological outcome after TaTME is decrease in involvement of CRM. The average involved CRM rate after laparoscopic abdominal rectal resection in large randomised trials including TME is 6–8% [2–10]. This systematic review shows an involved CRM rate of 4.3% after TaTME. CRM is a most significant prognostic factor for local recurrences and might relate to the expertise of the surgeon. Positive distal resection margins were found in 0.3% of the patients. These objective surgical quality measurements compare favourably to the published surgical laparoscopic rectal cancer studies, especially since the majority of the data are obtained from mid and low rectal cancer, whereas the large laparoscopic trials include low, mid and high rectal cancers [2–10]. It is debatable whether these data from cohort series can be compared to an audited clinical (randomised) trial. Nevertheless, TaTME potentially shows benefits over the laparoscopic TME regarding these oncological outcomes.

In the major trials investigating laparoscopic surgery for rectal cancer, the local recurrence rate for mid and low rectal cancer is approximately 5% after three years [6–8, 11]. The local recurrence rate as shown in this systematic review is 4%. However, this number is likely an underestimation due to the inadequate length of follow-up (18.9 months) and inadequate number of studies reporting follow-up. Interestingly, the involved CRM rate was similar to the local recurrence rate. Concern regarding intraluminal spread or other unknown factors exist, but it has to be noted that due to inadequate numbers and lack of long-term oncological follow-up preferably from randomised data no conclusions can be drawn.

This systematic review evidently shows a relationship between case volume and outcomes. Higher-volume centres have better outcome compared to small volume centres. Although statistical significance could not be obtained since lack of original data including standard deviations, a clear trend is visible. Operative time and conversion rate were lower, and the use of two simultaneous teams for the abdominal phase and the transanal phase during TaTME was performed more frequently in the high-volume centres compared to small volume centres. More interestingly, both quality of the resection and post-operative outcome were better in high-volume centres. However, an actual learning curve could not be extracted from the included studies, as a proficiency curve has yet to be determined and individual rates of series and outcomes were unavailable.

These data reflect the relative difficulty of the procedure requiring multiple skills including single-incision laparoscopic surgery (SILS) technique and two-team operating. As is known from colon surgery and oesophageal surgery, higher volume is associated with better outcomes [25]. For TaTME, this equation seems equal to the other difficult procedures. Although the quality of the data is non-randomised, this difference seems valid and calls for education, training and proctoring in order to have a safe introduction of the TaTME technique. A well-designed trial in which surgical quality assurance is an essential component should be ideal to evaluate the potential benefit of the TaTME technique. Before entering the trial, a surgeon should be trained and proctored and its surgical performance should be objectively monitored in order to exclude underperformance within the trial.

A major limitation of the available data is the lack of randomised evidence. Current cohort data are the result of the pioneers. The TaTME technique is technically demanding of both surgeon and team and requires a learning curve. Another limitation of this and previous systematic reviews is that the included studies are heterogeneous concerning clinical and tumour characteristics, surgical details and reporting of complications. Therefore, comparison of these studies and outcomes of this review should be carefully interpreted. Moreover, most of the studies include same patients in different reports. Abstracts, congress supplements and other unpublished data were not included with the aim to exclude major bias in contrast to previous published reviews. Furthermore, in the low- versus high-volume analysis, all (partial) duplicate publications were excluded. Nonetheless, most papers represent a small number of patients and high-quality studies are lacking. In the absence of published data concerning a learning curve or number of cases to achieve proficiency, we choose to use a cut-off point of 30 based on the traditional rectal surgery and agreement of the consensus group. We realize that this subanalysis is prone to bias. Furthermore, the majority of the studies exclude tumours with ingrowth in surrounding tissues. Especially, rectal cancer surgery in patients with T4 tumours is challenging and needs improvement, in specific regarding the quality of the resected specimen. Finally, adequate follow-up period of most studies is lacking and hampering any firm conclusions about long-term outcome.

Nevertheless, even at this early stage of implementation of the TaTME technique, it is important to provide a critical overview of the experience and outcomes of the procedure worldwide and especially to highlight the technical difficulty and possible hazardous aspects of TaTME. The TaTME consensus group has stated that at least 14 procedures a year have to be performed in order to assure optimal quality of the procedure [56]. To ensure save implementation and consistency in surgical quality, several TaTME expert centres across Europe and the USA provide training workshops and facilitate proctoring of the technique. Within the context of a future randomised controlled trial, quality assurance of this new technique seems of paramount importance.

In conclusion, TaTME is a potentially advantageous procedure for mid and low rectal cancer. Despite the current data available is mainly based on expert centres, considerable morbidity has been reported. In order to avoid unwanted negative outcome associated with widespread uncontrolled use of this novel technique, quality assurance and controlled safe implementation seem essential. TaTME has high potential; however, extensive evaluation in a well-designed multicentre randomised trial is needed to come to unequivocal conclusions.
